# The Feasibility and Acceptability of The Girls Peer Activity (G-PACT) Peer-led Mentoring Intervention

**DOI:** 10.3390/children5090128

**Published:** 2018-09-19

**Authors:** Michael B. Owen, Charlotte Kerner, Sarah L. Taylor, Robert J. Noonan, Lisa Newson, Maria-Christina Kosteli, Whitney B. Curry, Stuart J. Fairclough

**Affiliations:** 1 Movement Behaviours, Health and Wellbeing Research Group, Department of Sport and Physical Activity, Edge Hill University, St Helens Road, Ormskirk, Lancashire L39 4QP, UK; Charlotte.Kerner@brunel.ac.uk (C.K.) sarah.taylor11@go.edgehill.ac.uk (S.L.T.); robert.noonan@edgehill.ac.uk (R.J.N.); maria-christina.kosteli@edgehill.ac.uk (M.-C.K.); stuart.fairclough@edgehill.ac.uk (S.J.F.); 2 Department of Life Sciences, Brunel University, London UB8 3PH, UK; 3 Natural Sciences and Psychology, Research Centre for Brain and Behaviour, Liverpool John Moores University, Liverpool L3 5AF, UK; l.m.Newson@ljmu.ac.uk; 4 Wellbeing and Public Health, Cornwall Council, Truro TR1 3AY, UK; whitney.curry@cornwall.gov.uk

**Keywords:** adolescents, girls, school, physical activity, feasibility, acceptability, intervention, peer-led, mentor, leader

## Abstract

Enjoyment of physical activity (PA) is positively correlated with PA engagement. The inclusion of peers has been found to increase the likelihood of PA enjoyment in youth. Peer-led strategies, incorporating peer networks in the intervention delivery, is relatively underused and consequently understudied in school-based PA interventions. The purpose of this investigation was to evaluate the feasibility and acceptability of the novel Girls Peer Activity (G-PACT) peer-led mentoring intervention. Two-hundred and forty-nine Year 9 adolescent girls (13–14 years old) from three mixed-sex secondary schools located in West Lancashire, North-West England were invited to participate in the G-PACT project. The study employed a novel approach by using a three-tier model, including (Tier 1) Mentors (undergraduate students), (Tier 2) Leaders (Year 9 girls selected by teachers), and (Tier 3) Peers (whole Year 9 cohort). Mentors delivered a series of educational and leadership training to the Leaders in each respective school who then disseminated this information to their Peers and encouraged them to engage in more physical activities. Eight focus groups were conducted with Leaders (n = 40), 28 focus groups with Peers (n = 185), two focus groups with Mentors (n = 6), and three interviews with teachers (n = 4). Thematic analysis was used to analyze the pooled data and identify the key themes. The study found that the G-PACT intervention was feasible and acceptable for adolescent PA Leaders and their Mentors. The relationship between Leaders and their Peers required refinement to improve the communication processes to increase Peer engagement in the G-PACT project.

## 1. Introduction

Physical activity (PA) enjoyment is positively correlated with PA engagement [1]. Interventions aiming to increase moderate to vigorous PA (MVPA) in children and young people, which included the recruitment of friends, have been found to increase enjoyment of MVPA [2]. Adolescents with physically active friends are more likely to be physically active and spend less time engaging in sedentary behaviors such as, TV viewing [2]. 

Objectively measured accelerometry data from multiple continents suggests that among 5–17-year-old, only 9% of boys and 1.9% of girls achieved the recommended 60 min of MVPA [3]. In 2016, 22% of English children and young people (aged 5–15 years) met the recommended 60 min of MVPA guidelines [4]. However, only 16% and 9% of girls aged 11–12 and 13–15 respectively met to the guidelines [4]. Thus, adolescent girls in England have been highlighted as a group at risk to declining PA levels.

There have been calls to find new ways to support and encourage sustained engagement in PA for adolescent girls [5]. One strategy that is relatively underused and consequently understudied in school PA interventions is the use of peer-led approaches [6,7,8,9]. Peer-led involves similar aged peers [10], interacting with and motivating their classmates to initiate, continue and sustain positive behavior [11,12]. Previous interventions using the peer-led model to increase PA have shown the potential to increase girls’ MVPA [6,9,11,13]. The PLAN-A intervention (Peer-Led physical Activity iNtervention) incorporated a novel school peer-led approach, which included the nomination and training of peer-supporters, followed by a 10-week informal PA message diffusion period [13]. Targeting adolescent girls, the PLAN-A intervention was found to be feasible, showed promise to positively affect girls’ PA levels, and warranted further investigation to assess the effectiveness and cost-effectiveness of the intervention. The Go Active (Get Others Active) 12-week intervention [9] targeted both adolescent boys’ and girls’ PA levels, using tiered leadership system [6]. Mentors (older adolescents within the school) and peer-leaders (within each Year 9 class) encouraged students to try new physical activities each week, which was found to be feasible [6]. Future trials in the GoActive project will aim to assess the effectiveness and cost-effectiveness [9] of this approach and a mixed-method process evaluation [14] to explore experience in, and implementation of the Go Active intervention.

The GLAMA (Girls! Lead! Achieve! Mentor! Activate!) intervention incorporated a cross-age component to develop and foster leadership skills in Year 10 girls (15–16 years old) so they were capable of leading a group of Year 7 girls (12–13 years old) in a range of physical, cognitive and team focused activities [10]. The GLAMA process evaluation identified several school level barriers which impeding the planned delivery of the intervention. These included the structure of the curriculum, timetabling, pressure to meet curriculum and assessment content, lack of support for new initiatives, multiple programs already running within the school, time allowances for teachers, and appropriate training for teachers [10]. That said, the intervention was well received by peer-leaders, peers and teachers, leading researchers to recommend the cross-age intervention to be implemented over a longer duration [10].

Novel peer-led interventions require appropriate investigation to assess their feasibility and acceptability before piloting and upscaling. Feasibility studies are increasingly undertaken to explore unanticipated factors in the development and implementation of complex interventions and are recommended to inform piloting and upscaling [6,15,16]. The implementation process in complex interventions can be affected by multiple unexpected factors [17], which can influence effectiveness of the interventions [18]. Appropriate and well conducted mixed methodological research can however, make an important contribution to assess the feasibility and acceptability of complex interventions [15], and highlight factors which can influence implementation. The purpose of this investigation was to evaluate the feasibility and acceptability of the Girls Peer Activity (G-PACT) peer-led mentoring intervention [19]. The study aimed to assess if a school peer-led mentoring intervention with an educational component was (1) feasible and acceptable for adolescent PA Leaders; (2) feasible in communicating PA messages with adolescent girls (13–14 years old); and (3) feasible and acceptable in the secondary school setting when used in combination with additional PA opportunities.

## 2. Materials and Methods

The G-PACT project was a feasibility and acceptability study of a novel three-tier peer-led mentoring intervention. The G-PACT project contained three distinct phases; *phase one* was an exploration study to assess the current school PA practices and the needs of adolescent girls, *phase two* was a quantitative assessment of the G-PACT intervention to assess the impact on girls’ PA levels [19], and *phase three* (detailed below) was a qualitative assessment of the feasibility and acceptability of the G-PACT project. Trial registration number: ISRCTN51511240.

### 2.1. Participants

Two-hundred and forty-nine Year 9 adolescent girls (13–14 years old) from three mixed-sex secondary schools located in West Lancashire, North-West England were invited to participate in the G-PACT project. The three secondary schools were in areas with similar socio-economic characteristics, based on the UK government Indices of Multiple Deprivation (IMD) deciles (UK decile 6 or 7; decile 1 represents the most deprived 10% of areas nationally [20]). The IMD is a UK Government measure comprising seven areas of deprivation, including income, employment, health, education, housing, environment, and crime.

### 2.2. Recruitment

A purposeful sampling strategy was employed to recruit schools [21]. With input from the research team, feedback from physical education (PE) teachers, and *phase one* G-PACT data, a new 7-week school peer-led mentoring intervention was developed. The PE teachers in each respective school used convenience sampling during PE classes to recruit Year 9 girls (13–14 years old) to participate in the intervention. Ethical approval was granted from the Faculty of Arts and Sciences Research Ethics Committee at Edge Hill University (SPA-REC-2016-340).

### 2.3. Intervention

The G-PACT intervention has been described in detail previously [19]. The intervention incorporated a peer-led mentoring model underpinned by Social Cognitive Theory (SCT) [22,23,24] and Self-determination Theory (SDT) [25]. The study employed a novel approach by using a three-tier model, including (Tier 1) Mentors (undergraduate students), (Tier 2) Leaders (Y9 girls selected by teachers), and (Tier 3) Peers (whole Year 9 cohort). The Mentors delivered a series of weekly leadership and educational sessions to the group of Leaders, in each respective school, which incorporated information on: PA; health, motivation; barriers to PA; ideas on how to increase PA; ideas how to encourage Peers to be more physically active; and social support for their role.

The intervention was implemented for 7 weeks, which was due to school timetabling and data collection materials (accelerometer) restrictions. During the 7-week period all three schools received the same peer-led mentoring program with an educational component. In addition, two of the schools received an after-school PA component. School one (*Class*) received a fitness class-based after-school component, school two (*Choice*) received a choice-based component, girls could self-select activities, and school three (*No Club*) did not receive a PA component until cessation of the intervention. The after-school component ran alongside the other intervention components, once a week for 6 weeks, and were open for Peers to attend. During week 1 of the intervention, Leaders were invited to visit a university campus for a half-day workshop. The Leaders were informed of their roles in the project and discussed with their Mentors the best way to fulfil their roles and responsibilities within their respective schools. Through informal discussions, the Leaders were encouraged to disseminate the information they had learnt through their educational sessions to their friends and Peers. The Leaders were also asked to help to design information leaflets and posters to encourage more PA, including advertising the new after-school PA opportunities where appropriate. This peer-led approach was used as social influence through friends and peers and is crucial for adolescents to attain the best health behaviors in the transition into adulthood [26].

### 2.4. Measures

All consenting girls (Leaders and Peers) who completed post-intervention (post-INT) data collection (week 8), as part of the G-PACT project [19], participated in focus groups to assess the feasibility and acceptability of the intervention. Eight focus groups were conducted with Leaders (n = 40), 28 focus groups with Peers (n = 185), two focus groups with Mentors (n = 6), and three semi-structured interviews with teachers (n = 4). Across all three schools, each data collection session at week 8 lasted approximately 1 h in duration. It was anticipated that all data collection measures and analysis would take 6 months to complete.

#### 2.4.1. Qualitative

##### Semi-Structured Focus Groups (Peers, Leaders, and Mentors)

The focus groups were conducted to elicit girls’ experiences of the 7-week intervention. The focus group method was used with adolescent girls rather than face-to-face individual interviews as girls were expected to feel more confident and comfortable in a group in their own environment [27,28,29,30]. Focus group methodology enabled open discussion and allowed the girls to build on each other’s comments [27,28]. The focus groups were semi-structured so that the facilitator could ask probing questions around the pre-defined topics, keep the discussions relevant to the study aims, and to ensure consistency across focus groups [30]. All three focus group types (Peers, Leaders, and Mentors) were conducted using the same research protocol and followed a pre-defined schedule containing questions appropriate to their role in the G-PACT project (Supplementary 1). For example, questions for Peers related to interactions with Leaders, intervention after-school activities, barriers to engagement and overall experiences of the intervention.

The girls were grouped by role in the G-PACT project; therefore, Leaders and Peers participated in separate focus groups. The focus groups for Leaders and Peers were conducted at the school sites. Trained female research assistants (18–24 years old) conducted the Leaders and Peers focus groups. Focus groups were conducted with all available Peers and Leaders (rather than a sub-sample), to assess the feasibility and impact as they were the primary target of the intervention. Each focus group with Peers and Leaders contained 5–8 girls, and lasted between 10 and 22 min, with an average duration of 15 min. The focus group durations were constricted due to the time limitations within the schools (i.e., curriculum time). Mentor focus groups were conducted 2-weeks post-intervention. Mentor focus groups were conducted at the university site by the lead author and a research assistant. The two Mentor focus groups were more in-depth, contained three Mentors each, and lasted 1 h and 14 min and 1 h and 20 min, respectively. 

##### Interviews (Teachers)

Interviews were conducted with teachers to elicit their perceptions of the feasibility of the intervention and its subsequent impact on adolescent girls. The teachers were asked about their thoughts and observations of the G-PACT project including, the Leaders and Peers interactions. Teacher interviews were conducted 2-weeks post-intervention. Teacher interviews were conducted at their respective school sites by the lead author. The interviews with teachers from *Class* and *No Club* school were conducted on a 1:1 basis. For the convenience of the teachers in the *Choice* school, the interview was conducted on a 1:2 ratio as they held a shared role, and both wished to participate. The interviews followed a semi-structured interview schedule (Supplementary 1), this allowed for discussion on the key topics but also allowed flexibility to explore important areas as they arose. The interviews lasted 25–35 min, averaging 31 min.

#### 2.4.2. Quantitative

##### Questionnaire (Peers)

Preceding the focus groups, Peers were asked to complete a short questionnaire about their experiences in the G-PACT project. The questionnaire contained four closed-questions (‘yes/no’ responses) relating to; interaction with PA Leaders, additional PA with friends, PA information received from Leaders and new PA information learnt. The questionnaire also included one open-ended question asking the Peers to provide some feedback about their experiences in the G-PACT project over the past 7-weeks. This questionnaire method was used to provide Peers with an additional opportunity to discuss their experiences of the G-PACT project [29]. The questionnaire method was used to support the focus group responses and provided data triangulation [31,32]. 

### 2.5. Analyses

#### 2.5.1. Qualitative

##### Focus Groups and Interviews (Peers, Leaders, Mentors and Teachers)

Audio-recordings from the focus groups and interviews were transcribed verbatim. To ensure anonymity, all names were removed and replaced with pseudonyms. An inductive and data driven analytical strategy was used to identify and discuss the salient themes repeated across and within the transcripts [33]. Thematic analysis has been utilized for this study as it allows for the identification of patterns and meaning across a dataset and provides a flexible approach needed in feasibility studies [34]. An inductive analysis, rather than a deductive or mixed analysis, allowed for a ground-up approach and the exploration of unanticipated findings, which are common in feasibility studies [33].

**Figure 1:**
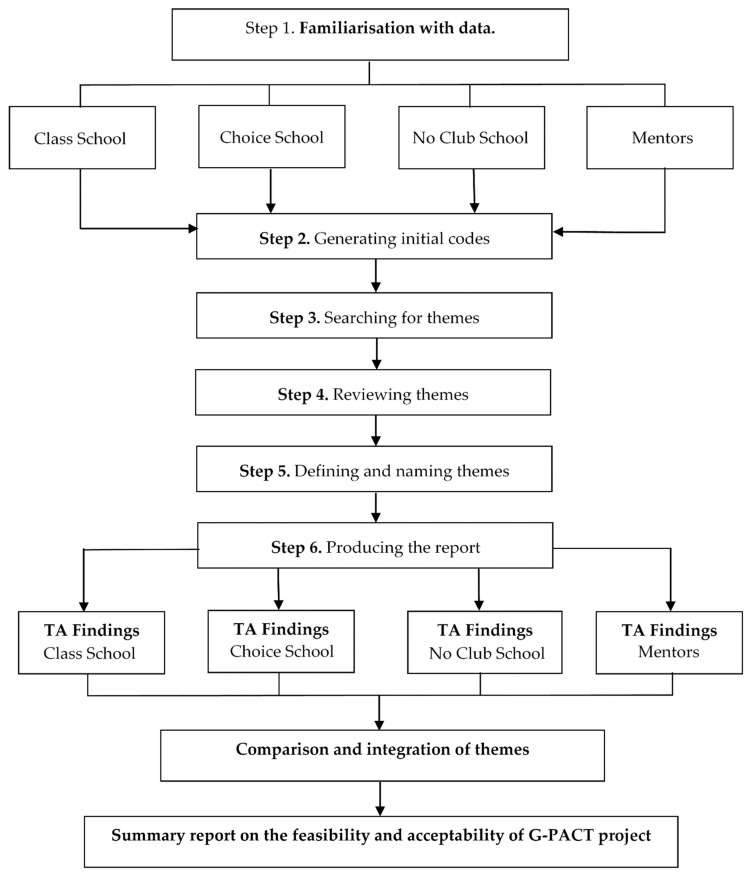
The application of step-by-step thematic analysis (TA) [34].

Inductive thematic analysis of the data was completed, by the lead author, using the step-by-step guide set out by Braun and Clarke [34] (Figure 1). Due to the time restrictions in the school setting, in-depth focus groups were not possible. All data collected from all sources were analyzed.

The separate data sources (focus groups, interviews, open-ended questionnaires) were pooled together by intervention group (*Class*, *Choice* or *No Club*) and a mixed analysis approach was taken for complimentary purposes (Figure 1). This allowed for comparisons within and across data sources for each intervention group [31,32]. The data was analyzed individually by intervention group (school). Additionally, the data from the Mentors was analyzed separately to the individual intervention group data, as the Mentors were involved across all intervention groups (Figure 1). Following this, the themes from each analysis were compared and integrated to provide an overall analysis of the feasibility and acceptability of the G-PACT project. The pooled data resulted in 877 pages of raw transcription data (Times New Roman, size 12, double spaced).

All codes and emergent themes were checked by a second author (M.-C.K) to ensure consistency of coding. Any disagreements were discussed (M.B.O. and M.-C.K) until a 90% agreement level was reached. After finalizing themes, quotes that were deemed to best represent each theme were then selected to illustrate the wider views of the specific population. Triangulation of data was achieved through comparison of the questionnaire, focus group and interview data [31,32]. The questionnaires were used to support the focus group data with Peers and Leaders regarding the feasibly of the intervention design (i.e., Peers interaction with Leaders, additional PA with Leaders and what the girls had learnt during the intervention period). The open-ended aspect of the questionnaire also provided the girls with an opportunity to provide feedback on their experiences in the project, which were used to assess the acceptability of the intervention. To ensure methodological rigor, credibility, and trustworthiness [32,35], the focus groups, interviews and open-ended questionnaire responses were independently reviewed by a third author (R.N.), and were then cross-examined against the data in reverse, from the themes to the data sheets [36]. Any disagreements were discussed between the three authors (M.B.O., R.J.N., and M-C.K.; 90% agreement).

#### 2.5.2. Quantitative

##### Questionnaire (Peers)

Frequency counts and percentages were calculated by school for the four ‘yes/no’ questions. The text from the open-ended question was added to the main analysis described below.

## 3. Results

### 3.1. Questionnaire

The responses were not consistent across schools for some elements (Table 1). For example, in the *Choice* school 62% of Peers received information about PA from friends or classmates over the previous 7-weeks compared to just 34% and 32% in the Class and *No Club* school, respectively. The *Choice* school reported the highest percentage of Peers who had spoken to a PA leader. Whereas, the *Class* school had the highest number of Peers who reported doing additional PA with friends or classmates over the previous 7-weeks. 

### 3.2. Thematic Analysis

#### 3.2.1. Class School Summary

The main overarching themes arising from the data for Class school were Engagement, Mentor Rapport, Leadership Selection and After-school Club Advertising. Not all the girls were engaged in the G-PACT project, as some of the Leaders did not fulfil their roles as PA Leaders, and some only engaged their close friends. “We can’t get involved if there not allowing us to get involved” (Peer). This was mainly due to the leadership selection. “We selected students that were potentially Leaders, but not sports Leaders, and then I’ve got the wrong mix of students,” (Teacher). Although some of the chosen Leaders were confident and influential, Teachers believed they were not suited to the PA leadership role. 

The Mentors struggled to build rapport with certain Leaders and this contributed towards the Leaders’ engagement and subsequent interaction with their Peers. The Mentor to Leader relationship showed feasibility but this was reliant on the Leaders being engaged in their roles and the Mentors ability to build rapport. The after-school club was well received by the girls but a ‘taster’ session during PE lessons would have been useful to advertise the club and engage harder to reach girls at the start of the after-school club program. “Their friends can tell them what it’s like, but a lot of girls at that age don’t have the confidence, offering the girls some taster sessions in PE would have probably been a really big winner” (Teacher). The additional PA component was a feasible and acceptable method in combination with the peer-led mentoring model to, Peers, Leaders and Teachers.

#### 3.2.2. Choice School Summary

The overall themes originating from the Choice school data were Leadership Role Clarity, Leader Engagement, Peer Engagement and After-school Club Environment. The Leaders clearly understood their roles and responsibilities. “how to communicate with other people, how to motivate them…and that you need to make sure you’re doing about 60 min of activity a day and that will help you increase your fitness too” (Leader). There was a strong link between Mentors and Leaders, demonstrating that this relationship was feasible and acceptable. The Leaders were committed to their roles and developed their leadership skills as the G-PACT project progressed. “They actually called a Year 9 assembly, and the Leaders actually stood up and delivered stuff on how to get more active and advertised the new club” (Mentor). However, there was a gap in communication between the Leader and some of Peers. “A lot of people (Peers) are just like nah can’t be bothered… it was hard to motivate people to come to the new club. Like you have to try and persuade some people to come, bribe them (laughs)” (Leader). 

The Peers enjoyed the novelty of receiving information from their friends “They (Leaders) were giving out information leaflets out about a new after-school club” (Peer) but, the Leader-to-Peer communication process needs refinement to improve effectiveness and coverage of the messages to all Peers. Peers who engaged in the new after-school club program enjoyed the novelty and choice provided within the sessions. “Because they had a choice of like new activities to do, that was more appealing to them” (Teacher). The new after-school program helped provide a positive motivational climate to support girls’ PA behaviors. “I liked it because it wasn’t so much exercise but it’s like fun exercise like you would look forward to it because you know you’re going to have a laugh but also doing exercise” (Leader). The additional PA opportunity was acceptable and feasible alongside the peer-led mentoring model as it provided Leaders with a school-based opportunity to encourage more PA within their friendship groups.

#### 3.2.3. No Club School Summary

There were two overarching themes emanating from the data from the No Club School, which were Leader Development and Friendship Groups. The Leaders developed their knowledge and understanding of PA and their leadership skills because of the G-PACT project. “I have learnt how to be more confident about leading a group of people in a sports activity. I have also learnt to help my friends get involved with activity. I have also learnt to encourage people more whilst doing activity and to get people to do more activity.” (Peer). The Mentor to Leader relationship demonstrated feasibility and acceptability of this intervention approach. “They were lovely … it was dead different because they’re more laid back, they understand where we’re coming from … it was a lot more informal … because the Mentors were younger as well and they loved sport like we do, so they could relate to us a lot more” (Leader). However, membership in a certain friendship group dictated Peers’ engagement in the project. Depending on what friendship group their Peers were part of; Peers were either engaged or unaware of the main G-PACT project components. There was a breakdown in communication between Leaders and the wider group of Peers. “Not everyone’s going to listen to you because if you don’t really know someone that well then they’re just going to be … not really listening to you. So if someone allocated us a time to talk about things people have to listen then it would be a bit different because then they’d actually take it in” (Leader). The Leader to Peers relationship was feasible; however, refinements to the process were needed to engage a wider group of Peers, beyond the Leaders’ close friends. The Leaders may have benefitted from additional PA opportunities to help promote greater PA engagement within their friendship groups. 

#### 3.2.4. Mentors Summary

There were four main themes arising from the Mentors focus groups: Time to Develop, Leader Selection, School Space, and Project Structure. The Mentors needed time to develop into their role, grow in confidence and build rapport with their Leaders. “Your confidence develops, you’re getting familiar with your role and things like that … the more and more you do it, the more confident you get” (Mentor). The Mentor to Leader approach was feasible and acceptable from the Mentors perspective, but the relationship would have benefitted from a greater amount of time to develop into the roles and build rapport. “It was just difficult to try and find a balance on how to be with them, it took time. You want to be fun, and you want to be friendly and nice, but you also want to make sure that they’re getting things done” (Mentor). The Leadership selection was important to the engagement of Leaders and Peers. Schools that selected appropriate Leaders who were willing to engage in project activities and were enthusiastic about their role benefitted most from the G-PACT project. This highlighted a vital element critical to the feasibility of this approach, the leadership selection of girls. Across the three schools, the provision of suitable spaces to conduct the intervention activities was insufficient. “We made it as fun as we could, but there was nothing that we could have done, because we didn’t have the space for it” (Mentor). The G-PACT project was feasible when conducted over a 7-week period but could have benefitted from minor amendments to the structure of the leadership and educational sessions. Moreover, the intervention may have been more effective over a longer duration to allow more time for Mentors and Leaders to develop into their roles.

#### 3.2.5. Comparison and Integration of Themes

There were themes identified in each school that closely related to themes from other schools or mentor data. The schools that selected Leaders who engaged in their roles and had a good understanding of their responsibilities benefitted most from the intervention. Influential Leaders in the Class school hindered the relationship building process between Mentors and Leaders and thus, negatively influenced the implementation of the intervention. Leaders in the Choice and No Club schools in comparison, were able to build strong relationships with their Mentors although rapport did take some time to develop. This may have been due to the personal qualities and engagement levels of the Leaders in the Choice and No Club schools. The Leaders who engaged with the leadership and educational training sessions developed their PA knowledge, confidence, and leadership abilities.

However, across all three schools there were breakdowns in communication between Leaders and the wider group of Peers. The Leaders’ communication with their Peers appeared to be dependent on associations with friendship groups. The Leaders in the Choice school were engaged and had a clear role clarity. When compared to the Class school, Leaders struggled to build rapport with their Mentors and teachers commented that the Leaders selected lacked the appropriate skills and quality to lead. This may have contributed to the poorer Peer engagement in the Class school compared to the Choice school.

For the two schools that received an after-school club component, these were generally well advertised by Leaders (posters and word-of-mouth) and subsequently well received by Peers and Leaders. The girls in the *Choice* school especially enjoyed the after-school activities as they had some autonomy over the choice of activity. Not having an after-school PA opportunity in the *No Club* school impeded the Leaders ability to encourage their Peers to engage in more PA during the intervention period. Overall, the intervention showed feasibility and acceptability for the relationship between Mentors and Leaders. The relationship between the Leaders and Peers to communicate PA messages requires refinement to improve effectiveness, but it is feasible. The peer-led mentoring approach alongside an after-school PA opportunity and was feasible and acceptable in a secondary school setting. 

#### 3.2.6. Refinements

As illustrated in Table 2. there are refinements to the G-PACT intervention that are applicable across schools. It is evident that the leadership selection method requires improvement. Leaders with influence over fellow peers should be considered carefully before selection. “We selected students that were potentially Leaders, but not sports Leaders, and then I’ve got the wrong mix of students, which meant that the ones that potentially we would have engaged became disengaged because some of the girls were too domineering and intimidating” (Teacher). Leaders with enthusiasm and a passion for leadership and a willingness to be involved in extra-curricular activities appeared to be best suited to the leadership roles in the G-PACT project. Taster Leadership sessions could be provided for the potential Leaders to assess their willingness to do the role and their suitability before giving them the responsibility and leadership training programme. These taster sessions would ensure that the Leaders have (1) more time to build rapport with Mentors; (2) greater ‘buy in’ from the start of the program, and (3) clearly understand their roles and responsibilities in-depth before starting the program. Additionally, it is apparent that across all schools there is a greater need for a clearer project identity or branding from the start and this is related to making the Leaders more identifiable to their Peers from the start of the intervention.

To increase the effectiveness of the program and ensure consistency across all participating schools, greater support for Leaders in their role is needed including: greater teacher involvement; more sessions during the school day; structured sessions whereby Leaders can directly communicate with the wider group of peers; and importantly a greater provision of PA opportunities to support peer engagement. “During form (registration) or something they should have gave us a time to talk to everyone because not everyone’s going to listen to you because if you don’t really know someone that well then they’re just going to be … not really listening to you. So if someone allocated us a time to talk about things people have to listen then it would be a bit different because then they’d actually take it in” (Leader). A longer intervention duration could be considered, and this would support the rapport development between Mentors and Leaders. These refinements would ensure that future programs of the G-PACT project do not encounter similar issues.

## 4. Discussion

The aim of the study was to evaluate the feasibility and acceptability of a school peer-led mentoring intervention designed to increase PA levels and reduce sedentary time of adolescent girls. The study found that the intervention was feasible and acceptable for adolescent PA Leaders and their Mentors but the relationship between Leaders and their Peers required refinement to improve the communication processes. The use of an additional PA component in combination with the peer-led mentoring approach was feasible and acceptable in a secondary school setting.

This intervention was informed by previous research, underpinned by theory (SDT and SCT) and was multi-component in nature, as recommended to increase adolescent girls’ PA levels [6,37,38]. The intervention builds on previous PA peer-led approaches with adolescents [6,8,9], incorporating university students as Mentors to provide guidance and support to PA Leaders. This study is one of the few to use a methodological approach that enabled researchers to capture data from all key stakeholders and the triangulation of data sources allowed for a comprehensive evaluation of both intervention feasibility and acceptability (enjoyment). The methodological approach and triangulation of data sources provides the study with a high degree of credibility and methodological rigor [36]. It was feasible to collect and analyze all data within a 6-month period. However, more time with the students to conduct the focus groups would have increased the depth to the qualitative accounts but this was restricted by timetabling and lesson time within the schools.

This intervention was implemented to be peer-led thus, it had a high degree of flexibility, including multiple interactions between Mentors, Leader and Peers. Therefore, this intervention can be classified as complex [18]. In accordance with the Medical Research Council guidelines for developing complex interventions this feasibility study achieved its primary aim to assess the acceptability of this intervention approach [16]. However, it is expected that for the development of complex interventions there should be a period of refinement before pilot testing to assess the likely rates of recruitment and retention of subjects, and the calculation of appropriate sample sizes. Numerous potential modifications were identified that need to be addressed before a pilot study phase. These included adapting the Peer engagement processes, leadership selection process, rapport building process for Mentors and Leaders and provision of PA opportunities.

The results identified a breakdown in communication between Leaders and the wider group of Peers. Leaders focused on delivering PA messages to their friendship groups, which highlights a limitation with this intervention approach that needs to be addressed in subsequent implementation. This was confirmed from data from teachers, Mentors, Leaders and Peers. If the Leaders only interacted with their friends and, where appropriate, encouraged them to engage in new after-school PA opportunities, this could have led to some Peers feeling isolated or unengaged. This relates to the Social Identity Theory [39,40], whereby it may have been difficult for some Peers to identify or relate to the Leaders and their friends. These Peers may have considered themselves as part of an out-group (a social group with which an individual does not identify) and therefore, unable to participate in the intervention components with the in-group (a social group to which an individual identifies as being a member) [39,40]. 

However, this disconnect between Leaders and Peers was less apparent in the *Choice* school. Leaders in the *Choice* school attempted to create one in-group through using their role to inform fellow Peers at an assembly about PA and the new after-school club program. The connection between Leaders and Peers may have been stronger in the *Choice* school due to the Leaders having greater autonomy over the intervention PA component. Autonomy was built into the intervention design through the SDT [41]. The PA component in the *Choice* school provided Leaders with the opportunity to engage with their Peers and suggest activities they may enjoy, creating a more inclusive in-group and more self-determined environment. Furthermore, supporting the Leaders, providing them with a degree of independence, and a structured opportunity to access all Peers may have positively influenced the social identification and comparison processes and subsequently increased group engagement [39,40]. 

Many previous peer-led PA interventions with other populations have included structured methods of delivery including, formal advice giving and leading educational classes [42]. By design, the current G-PACT intervention provided no formal structure for the Leaders to interact with their Peers. Similarly, others have used more informal methods to deliver health promotion messages in peer-led interventions [12,43]. The ASSIST (A Stop Smoking in Schools Trial) intervention adopted the Diffusion of Innovations Theory [44] and applied its concepts to informally diffuse stop smoking messages through social groups [12]. The intervention was effective in reducing smoking rates in adolescents compared to a control group. However, this informal approach may have not been wholly appropriate for the G-PACT intervention. The lack of intervention structure could have contributed towards a lack of clarity in the G-PACT intervention and communication breakdown in dissemination of key PA messages. A more structured formal approach, such as Leaders delivering structured sessions to peers in addition to the informal communications between friends warrants further exploration. Furthermore, future peer-led interventions with mentoring components should consider more extensive and structured guidance from the Mentors to the Leaders to reduce the ambiguity in their role and improve the intervention implementation.

Another possible contributing factor to the breakdown in communication was the Leaders’ personal attributes. The leadership selection process was highlighted across all schools as an aspect that could be refined to improve the intervention implementation. Although set criterion were provided to guide teachers’ selections of the Leaders (i.e., access to multiple peers and did not have to be perceived as sporty etc.), some were not engaged in the leadership role. Previous interventions applying peer-led approaches [12,43], and the planned PLAN-A [7] adolescent girls PA intervention, have used a peer nomination process to identify ‘influential’ peers who would be subsequently invited to be peer-leaders. However, the current G-PACT intervention found through the comparison of data sources, that some influential Leaders disrupted the leadership and educational program, did not engage in the role responsibilities and thus, may not be best suited for a PA leadership role. Leaders with a passion for Leadership, an interest in PA and a general enthusiastic attitude were well suited to the role.

Moreover, future leadership selection processes may consider investigating social networks before, during and post-intervention for peer-led approaches to assess the interactions between participants. Findings from a complex PA intervention using social network analysis found that those who exercised in pairs or a group maintained higher levels of PA than those who did not [45]. This provides support for peer-led approaches and the use of social network analysis to evaluate the intervention implementation and effectiveness. Similarly, the social network analysis may be a useful modification to the G-PACT intervention. This approach could be used to identify girls’ friendship networks, inform leadership selection and tailor the Leaders role to target their friendship networks only to improve implementation.

The Mentor to Leader approach was found to be feasible and acceptable. The leadership and educational workshops, underpinned by SCT [22,23,24] and SDT [41], introduced behavior change techniques to the Leaders [46] and provided leadership training to support their development. Health-mentoring interventions have been found to be an effective tool to increase knowledge and PA in clinical populations [47] but are understudied in youth. To the researcher’s knowledge, health-mentoring interventions have not been combined with a three-tier peer-led approach to increase adolescent girls’ PA behaviors. These structured leadership and educational workshops provided an opportunity for Mentors to build relationships and share experiences with their group of Leaders.

However, the Mentor to Leader approach could benefit from minor amendments. These include more time allocated to build rapport, greater detail on Leaders’ roles responsibilities, and greater surveillance on the Leaders’ engagement with peers, including an increased focus on barrier identification. Building rapport was commonly reported as a time consuming but an important process, Mentors felt that more time could have been dedicated to building this rapport to improve the mentor-leader relationship. A previous lifestyle-based ‘Go Girls’ intervention (i.e., PA, nutrition, and wellbeing) including a mentoring component with adolescent girls found that the Mentors relationship with students was critical to the success of the intervention [48]. The findings illustrated the importance of the Mentors’ ability to create a positive learning environment and sense of group belonging for the adolescent girls [48]. The G-PACT intervention was able to create a positive atmosphere for the adolescent girls, but it took time to develop. In addition, the structure of the leadership and educational sessions did not allow a specified period to develop rapport between Mentors and Leaders, which should be considered in the future. 

The refinements for the G-PACT intervention were solely based on findings from the current study. While previous interventions had similar peer-led elements [6,7,8,9,38,49], the mechanism of delivery and intervention content differs in each program. The G-PACT intervention delivery method had an extra component when compared to previous peer-led approaches. The incorporation of university students as mentors for adolescent leaders provided an additional level of support and guidance for the leaders from a relatable source. This third-tier to the intervention model added an extra layer of complexity to the intervention in terms of practicality however; the results and refinements suggest this approach was successful and well received. The peer-led approach is still a developing method to target youth PA behaviors. It is understudied in relation to the involvement of university students as mentors and the long-term impact of these approaches. Furthermore, adolescents are susceptible to changes relating to new influences and interests, which can affect behavior. Thus, ongoing peer research is warrant especially in relation to PA.

An additional PA component, implemented as a new after-school club, was feasible and acceptable. The *Choice* school after-school club, which provided girls with a choice over activities, was particularly well received. Providing choice and autonomy has been well established as a successful approach influencing youths’ PA enjoyment and engagement [25,50,51,52]. The SDT can be applied to understand the acceptability of this approach [41]. The choice element of the PA component created a sense of ownership and control over the activity. Combined with the PA component not being competence-based and accessible to all Year 9 girls this may have increased girls’ feelings of relatedness, competence, and autonomy. In turn, contributing to more self-determined forms of motivation and subsequent engagement in ‘their own’ after-school activity program.

Conversely, the *Class* school Leaders reported less independence and autonomy over the intervention. This may have been because the after-school PA component was prescribed by the research team based on previous exploration work. This lack of control and choice of activity may have decreased their sense of ownership and control, and subsequently decreased their motivation to disseminate information about the after-school club [53]. Nevertheless, when compared to the data sources from the *No Club* school, the additional PA component was an important aspect, which gave Leaders an opportunity to encourage their Peers to engage in more PA. Moreover, multi-component PA interventions have been found to be more successful with adolescent girls [37,54,55]. Future implementation of the G-PACT intervention should at minimum, contain an additional PA component, preferably a choice-based component providing greater autonomy [41].

### Strengths and Limitations

A strength of the study was the triangulation of data, collecting perspectives from multiple sources, and using multiple methods to assess the intervention feasibility including conducing focus groups with all available Peers. This provides the results with greater credibility, dependability, and trustworthiness. The qualitative methodology allowed for the collection of contextual information to support the assessment of the feasibility and acceptability of the G-PACT intervention [15]. The study was innovative by way of its design, incorporating university students as mentors and potential role models within a three-tier peer-led mentoring model. The use of older Mentors with an interest in and knowledge of PA provided Leaders with a relatable role model. The intervention was underpinned by multiple theories as recommended for intervention implementation [56] and effectiveness [37,54,57]. 

A limitation of the study is that some of the leadership and educational sessions for the Leaders were delivered within the school day and others after school. The pragmatic nature of the intervention, school timetable constraints, and availability of Leaders dictated that these sessions required a degree of flexibility, which meant that their timing could not be standardized between schools. However, this pragmatic approach demonstrated feasibility of implementing this type of intervention and allowed schools intervention flexibility, which is important in fluid environments such as schools [58]. Additionally, school timetable constraints precluded a greater period being made available to conduct the focus groups. The focus groups were short, which may have influenced quality and depth of the discussions, but they were conducted with all key stakeholders directly related to the intervention. Similarly, as focus groups can be challenging with this population, it is difficult to ensure that all participants feel comfortable to share their experiences and have the opportunity to do so [30,59]. Due to resource constraints, Mentors worked across all schools and were not blinded to the different intervention approaches. However, this provided the Mentors with an informed perspective to compare and contrast intervention approaches and implementation.

## 5. Conclusions

The combination and comparison of data sources from Peers, Leaders, Mentors, and teachers revealed that the G-PACT intervention was feasible and acceptable. However, the intervention requires a series of modifications and refinements before being developed into a pilot study. The data confirms that the link between the Mentors and Leaders was feasible and acceptable. The leadership and educational workshops were well received by the Leaders, supported their leadership development. There was a breakdown in communication between Leaders and their Peers, which requires modification to improve the effectiveness. The use of an additional PA component in combination with the peer-led mentoring approach was feasible and acceptable in a secondary school setting.

## Figures and Tables

**Table 1:** Peer questionnaire data for G-PACT project experiences and engagement.

Question	1. CLASS n = 78	2. CHOICE n = 64	3. NO CLUB n = 49
Spoke to the PA Leaders	25%	46%	30%
Did additional PA with friends or classmates over the past 7-weeks	78%	68%	70%
Received information about PA over the past 7-weeks from friends or classmates	34%	62%	32%
Learnt something new about PA over the past 7-weeks	36%	50%	62%

Note. % = Percentage of respondents who answered ‘yes’ to question.

**Table 2:** Refinements to G-PACT intervention based on the thematic analysis, presented by school and mentor data.

Class School	Choice School	No Club School	Mentors
Taster PA session	Taster PA session	After-school PA component needed	Greater focus on Leaders engagement across all Peers
Engage all Peers with structured sessions so Leaders can access wider group	Engage all Peers with structured sessions so Leaders can access wider group	Engage all Peers with structured sessions so Leaders can access wider group	
Leadership selection revision	Assess friendship networks to access more peers	Assess friendship groups as key to intervention exposure	Leadership selection revision
	Longer intervention duration		Longer intervention duration
Clearer project identity from the start of project	Clearer project identity from the start of project	Clearer project identity from the start of project	
Greater provision of additional PA opportunities	Greater provision of additional PA opportunities		Greater provision of additional PA opportunities
Greater focus on rapport development between Mentors and Leaders	More time to develop rapport with Leaders at start of project	More time to develop rapport with Leaders at start of project	More time to develop rapport with Leaders at start of project
Make Leaders more identifiable to Peers	Make Leaders more identifiable to Peers	Make Leaders more identifiable to Peers	After-school PA component needed for all schools
During school day intervention sessions for leaders	More teacher involvement		Appropriate space provision in schools to run sessions

## Supplementary Materials

The following are available online at http://www.mdpi.com/2227-9067/5/9/128/s1.
