# Supplementary material for: The Feasibility and Acceptability of The Girls Peer Activity (G-PACT) Peer-led Mentoring Intervention
Source: Children (Basel). 2018 Sep 19;5(9):128. doi: 10.3390/children5090128 (PMC6162561; doi:10.3390/children5090128)
Supplement: Supplementary file 1 [file children-05-00128-s001.pdf]

The Feasibility and Acceptability of The Girls Peer Activity (G-PACT) Peer-led Mentoring Intervention

Michael B. Owen [1,*], Charlotte Kerner [1,2], Sarah L. Taylor [1], Robert J. Noonan [1], Lisa Newson [3], Maria-Christina Kosteli [1], Whitney B. Curry [4], and Stuart J. Fairclough[1]

[1] Movement Behaviours, Health and Wellbeing Research Group, Department of Sport and Physical Activity, Edge Hill University, St Helens Road, Ormskirk, Lancashire, L39 4QP, UK; sarah.taylor11@go.edgehill.ac.uk (S.L.T.); robert.noonan@edgehill.ac.uk (R.J.N.); maria-christina.kosteli@edgehill.ac.uk (M-C.K.); stuart.fairclough@edgehill.ac.uk (S.J.F.)
[2] Department of Life Sciences, Brunel University, London, UB8 3PH, UK; Charlotte.Kerner@brunel.ac.uk
[3] Natural Sciences and Psychology, Research Centre for Brain and Behaviour, Liverpool John Moores University, Liverpool, L3 5AF, UK; l.m.Newson@ljmu.ac.uk
[4] Wellbeing and Public Health, Cornwall Council, Truro, TR1 3AY, UK; whitney.curry@cornwall.gov.uk
* Correspondence: Michael.Owen@edgehill.ac.uk; Tel.: +44-016956-57344

Supplementary 1 – Table 1. Example Topic Guides

Group	Topic Area	Example Questions
Peers	Interactions with Leaders	Did you receive any handouts from the Leaders?<br>Did you know who the Leaders were?<br>How did the leaders interact with you? (where and when)<br>Did the Leaders encourage you to do more PA?<br>How did the Leaders communicate with you? (face to face, phone)
	School-based physical activities	If you did attend the afterschool club?<br>What are your experiences of the afterschool club sessions?<br>Did you do any additional PA with your friends or leaders?
	Data collection procedures	How did you find wearing the activity watches (accelerometers) for 7 days?
	Leadership role	How did you find your role as a PA leader?<br>Did you feel supported in your role?

Leaders	Interaction with Peers	How did you interact with your peers?<br>How did you friends / classmates respond to the information you were passing out?
	Leadership training	After the first session, did you understand what your role was as a Year 9 PA leader?
	Interaction with Mentors	What were your experiences of your leadership training sessions?<br>How did you interact with your Mentors who took the leadership training sessions?
	School-based physical activities	Did you attend the new after school club in your school?<br>How did you feel about the type of activity chosen for the after school sessions?
	Data collection procedures	How did you find wearing the activity watches (accelerometers) for 7 days?
Mentors	Mentor Role	Did you understand your role as a Mentor?<br>Did your training prepare you for your role?<br>Did you encounter any barriers?
	Interaction with Leaders	How did you find delivering the leadership sessions to the Leaders?<br>How did you interact and engage with the Leaders?<br>How did the Leaders respond to the training?
	School experiences	What are you experiences of working in the school setting as part of this project?<br>Was the space provided in the school suitable for your planned sessions?<br>Did you interact with the school teachers?
Teachers	Project experience	What is your perspective on how the PA project was implemented within your school over the last 2 months?
	Leadership Selection	How did you find the leadership selection process?<br>Do you think this could have been improved?<br>What impact do you think the project had in your school?

	Leaders and Peers interaction	Do you think that the leaders fully understood their roles and responsibilities?<br>How did the Peers respond to the project?<br>How did the additional after-school clubs work?
	Project practicality	How much work was it for you personally to be involved in organising the project onto of your job demands?<br>What is you experiences of the girls wearing the activity watches (accelerometers)?
